# Hepatitis B virus X protein accelerates the development of hepatoma

**DOI:** 10.7497/j.issn.2095-3941.2014.03.004

**Published:** 2014-09

**Authors:** Xiao-Dong Zhang, Yuan Wang, Li-Hong Ye

**Affiliations:** ^1^Department of Cancer Research, ^2^Department of Biochemistry, College of Life Sciences, State Key Laboratory of Medicinal Chemical Biology, Nankai University, Tianjin 300071, China

**Keywords:** Hepatocellular carcinoma (HCC), hepatitis B virus (HBV), HBV X protein (HBx protein), hepatocarcinogenesis

## Abstract

The chronic infection of hepatitis B virus (HBV) is closely related to the occurrence and development of hepatocellular carcinoma (HCC). Accumulated evidence has shown that HBV X protein (HBx protein) is a multifunctional regulator with a crucial role in hepatocarcinogenesis. However, information on the mechanism by which HBV induces HCC is lacking. This review focuses on the pathological functions of HBx in HBV-induced hepatocarcinogenesis. As a transactivator, HBx can modulate nuclear factor kappa-light-chain-enhancer of activated B cells (NF-κB) and transcription factor AP-2. Moreover, HBx can affect regulatory non-coding RNAs (ncRNAs) including microRNAs and long ncRNAs (lncRNAs), such as miRNA-205 and highly upregulated in liver cancer (HULC), respectively. HBx is also involved in epigenetic modification, including methylation and acetylation. HBx interacts with various signal-transduction pathways, such as protein kinase B/Akt, Wnt/β-catenin, signal transducer and activator of transcription, and NF-κB pathways. Moreover, HBx affects cellular fate by shifting the balance toward cell survival. HBx may lead to the loss of apoptotic functions or directly contributes to oncogenesis by achieving transforming functions, which induce hepatocarcinogenesis. Additionally, HBx can modulate apoptosis and immune response by direct or indirect interaction with host factors. We conclude that HBx hastens the development of hepatoma.

## Introduction

Hepatocellular carcinoma (HCC) is one of the most common malignant cancers worldwide and ranks the third in annual cancer mortality rates. The chronic infection of hepatitis B virus (HBV) has been identified as a major risk factor in the development of HCC, accounting for 55% of cases worldwide; 80% or more of such cases are found in the eastern Pacific region and sub-Saharan Africa, which are the areas with the highest tumor incidence^1^. The mechanisms underlying HBV-induced malignant transformation remain ambiguous, but evidence has suggested that HBV X (HBx) protein serves a crucial function in the pathogenesis of HCC^2^. In this review, we focused on the molecular mechanisms of HBx in HCC development.

## *HBV X* gene and HBx protein

The HBV genome is circular with partly double-stranded DNA. The long (minus) strand is approximately 3,200 bases in length and contains four open reading frames (ORFs), namely, S, C, P, and X. *HBx* is the smallest gene of the four partially overlapping ORFs of HBV. The genome comprises 452 nucleotides, which encode a 154-amino acid regulatory protein with a molecular mass of 17 kDa^3^. HBx is the designated name of the gene and protein because the amino acid sequences failed to show homology with known proteins. HBx protein is highly conserved among the different subtypes of the virus and is common to all mammalian members of the *Hepadnaviridae* but not to avian viruses^4^. The HBx protein is present in the cytoplasm and, to a lesser extent, the nucleus of hepatocytes.

The enigmatic HBx protein is a transactivator that can activate various viral and cellular promoters and enhancers. HBx protein fails to bind directly to DNA, and its transcriptional activity is mediated via protein-protein interaction. The transactivation function of HBx may be exerted in the cytoplasm via signaling pathways and in the nucleus via DNA-binding proteins. HBx transcriptional activity is necessary for viral replication^5^. A high number of transcriptional factors [such as nuclear factor kappa-light-chain-enhancer of activated B cells (NF-κB) and AP-2], which directly interact with HBx, were recently identiﬁed^6^.

The clinical relevance of HBx begins with the integration of HBV DNA into the genome of the hepatocytes of chronic HBV carriers. The *HBx* gene is usually conserved in the integrant and is often found in the malignant hepatocytes of chronic HBV carriers. Our groups identified the *HBx* gene inserted into the upstream of a 20-bp *Alu* core sequence with a secondary deletion of 382 to 465 bp at its 3' end, followed by subtelomeric DNA; *HBx* gene-integration causes the recombination of HBx/*Alu* core sequence/subtelomeric DNA in the host genome^7^. HBx transcripts accumulate in the hepatocytes; the HBx induction in the hepatocytes seems to alter the expression of many genes involved in signal transduction pathways, cell cycle control, metastasis, transcriptional regulation, immune response, and metabolism^8^. Recently, Qiu *et al*.^9^ analyzed the differentially expressed genes between the LO2–HBx and LO2-vector control cells using microarray analysis, and the results were validated using quantitative real-time PCR. HBx upregulates 456 genes and downregulates 843 genes. These initial cellular targets of HBx provide a starting point in mapping the HBx mechanisms of action during the progressive development of HBx-mediated hepatocarcinogenesis.

## Mechanisms of HBx-induced hepatocarcinogenesis

### HBx and regulatory non-coding RNAs (ncRNAs)

The perspective on gene regulation in biology has mostly focused on the protein-coding genes via the central dogma of DNA→mRNA→protein. However, with the development of new techniques, such as high-resolution microarray and massively parallel sequencing, a minimum of 90% of the human genome has been actively transcribed into ncRNAs, and less than 2% of the genome sequences encode proteins^9^. According to their size, ncRNAs are divided into two groups, as follows: small ncRNAs (<200 nt) and long ncRNAs (lncRNAs). The interaction between HBx and ncRNAs is evident in HCC development.

#### HBx and small ncRNAs

Small ncRNAs include microRNAs (miRNAs), small-interfering RNAs (siRNAs), or PIWI-interacting RNAs. Among these small ncRNAs, miRNAs are the most detailed. MiRNAs are evolutionarily conserved noncoding RNAs (21 to 25 nt in length). These RNAs serve critical functions in gene expression regulation and multiple cellular processes, including proliferation, development, differentiation, and tumorigenesis. The alterations in miRNA expression have been observed in HCCs and are linked to the molecular pathogenesis of HCC because of their effect on the expression of crucial mRNAs. Depending on the target genes, miRNAs can function as tumor suppressor genes or oncogenes through mRNA cleavage or translational repression. HBx suppresses the p53-mediated activation of miR-148a, resulting in the upregulation of AKT and ERK and subsequent activation of mTOR to promote cancer growth and metastasis in a mouse model of HCC^10^. Recent studies reported that HBx suppresses the expression of miR-15b, which directly targets fucosyltransferase 2 and increases the levels of Globo H to enhance HCC cell proliferation^11^. miR-205 was downregulated in the liver of HBx-transgenic mice. HBx can abrogate the effect of miR-205 on tumor suppression in carcinogenesis^12^. The downregulation of miR-205 mediated by HBx remarkably increases the expression levels of acyl-CoA synthetase long-chain family member 1 (ACSL1), and its metabolite triglyceride levels are remarkably increased in HBx-induced liver cancer tissues, as shown in an HBx transgenic mice model^13^. HBx-transgenic mice have higher miR-224 expression than normal mice. Moreover, miR-224 promotes hepatoma cell migration and tumor formation by silencing its target gene *Smad4*^14^. HBx-mediated miRNA regulation serves a crucial function in hepatocarcinogenesis.

#### HBx and lncRNAs

LncRNAs are broadly defined as endogenous cellular RNA molecules that are longer than 200 nt. LncRNAs are poorly conserved and can regulate gene expression at various levels, such as during chromatin modification, transcription, and post-transcriptional processing. An increasing amount of evidence showed that lncRNAs serve critical functions in the development and progression of HBV-related HCC. For example, highly upregulated in liver cancer (HULC) is dysregulated in HCC. HULC expression level is positively correlated with HBx expression in clinical HCC tissues. Moreover, HBx can upregulate the HULC expression in human immortalized normal liver L-O2 cells and hepatoma HepG2 cells. The upregulation of HULC by HBx can promote the proliferation of hepatoma cells by p18 suppression^15^. Some studies have shown that HBx-transgenic mice show a specific profile of liver lncRNAs compared with wild-type mice; such profiles include the downregulated expression by HBx (Dreh; lncRNA–Dreh), which can inhibit HCC growth and metastasis *in vitro* and *in vivo* and functions as a tumor suppressor in the development of HBV-related HCC^16^. The lncRNA dysregulation, which is mediated by HBx, is closely related to tumorigenesis, metastasis, prognosis, or diagnosis of HCC; thus, lncRNA dysregulation serves an important function in hepatocarcinogenesis^17^.

### HBx and modiﬁcation of epigenetics

Early research on the pathogenesis of HBx-induced HCC and on other etiological forms of the tumor focused on the changes at genetic level. Recent studies showed that epigenetic changes, which inactivate tumor suppressor genes or chromosomal instability, serve an increasingly important function in hepatocarcinogenesis^18^. Epigenetic changes refer to the changes in gene expression mediated by mechanisms other than the alterations in the nucleotide sequence of that gene. Such alterations include aberrations in DNA methylation and histone modifications.

#### HBx and methylation

Reversible DNA methylation occurs at the sites of cytosine-guanine dinucleotides (CpGs) and can be generated by adding a methyl group to the carbon-5 position of cytosine. DNA methylation patterns are established by a family of DNA methyltransferases (DNMTs). The HBx-induced upregulation of DNMT activity is implicated in the aberrant methylation of CpG islands in some host genes that are involved in tumor suppression. Moreover, these DNMT-mediated methylated genes serve a function in cell cycle regulation, cell growth, dedifferentiation, invasiveness, apoptosis, immune escape, and xenobiotic metabolism, thereby contributing to HBV-related HCC onset and/or progression^19^. Recent studies showed that HBx can upregulate the expression of DNMT1 and DNMT3A by HBx transcriptional transactivation activity^20^. HBx induces the promoter hypermethylation of the genes with tumor-suppressing activity by upregulating the expression of DNMTs, as shown in the *p16^INK4A^* gene that negatively regulates cell cycle^21^. In addition, HBx significantly elevates the expression of genes by inducing the targeted promoter hypomethylation; these genes include several tumor promotion-related genes, such as retinal dehydrogenase 1, plasma retinol-binding protein precursor, and cellular retinol-binding protein I^22^.

#### HBx and acetylation

HCC usually occurs with aberrant acetylation, particularly histone acetylation. Such abnormal histone modifications perturb cellular gene expression, thereby disrupting normal cellular activities. Acetylation is controlled by histone acetyltransferases and histone deacetylases. HBx can promote HBV-induced HCC pathogenesis by inducing the histone acetylation of some tumor promotion-related genes. For example, HBx can strongly induce the expression of HDAC1 at transcription level, thereby enhancing the stability of hypoxia-inducible factor-1α, which may serve a critical function in the angiogenesis and metastasis of HBV-associated HCCs^23^. HBx can also promote the development of HBV-induced HCC by inducing the histone deacetylations of specific tumor suppression-related genes. Recent studies demonstrated that the HBx-mediated suppression of *cadherin-1* (*CDH1*) genes involves the recruitment of the transcription factor mSin3A/HDAC1 complex to the promoters of the Snail-binding sites in human *CDH1*^24^. HBx recruits HDAC1 to form a complex with Sp1 in a p53-independent manner and with deacetylated Sp1; subsequently, the binding of Sp1 to the targeted DNA during transcriptional repression is diminished^25^.

NAD-dependent deacetylase sirtuin-1 (SIRT1), a member of the class III histone deacetylases, is conserved during evolution of different species, from yeasts to humans. SIRT1 is implicated in several biological processes, including metabolism, cell division, differentiation, survival, and senescence^26^. In particular, SIRT1 mediates adaptation process to enhance the survival of an organism under cellular stress conditions, such as nutrient deprivation and oxidation. The presence of HBx attenuates the interaction between SIRT1 and β-catenin, thereby protecting β-catenin from the inhibitory action of SIRT1, which leads to anticancer drug treatment resistance^27^. The upregulation of SIRT1 expression or activity exposes Hep3B cells, which stably express HBx, to oxidation-induced apoptosis via the downregulation of c-Jun N-Terminal protein kinase activity^28^. These results suggest that SIRT1 serves an important function in protecting HBx-expressing HCC cells from oxidative stress, thereby promoting hepatocarcinogenesis.

### HBx and signaling pathway

#### HBx and Akt signaling pathway

As one of the most versatile kinase families, Akt (also known as protein kinase B) serine–threonine kinases critically regulate cell survival, proliferation, metabolism, and migration. The dysregulation of Akt kinases is frequently associated with human diseases, such as cancer^29^. Previous studies focused on the functional interaction between HBx and Akt; HBx can be a substrate of Akt kinase^30^. The molecular cooperation between HBx and Akt serves a key function in cell survival and oncogenic transformation. Researchers recently isolated two HBx isoforms from an HBV carrier. The first HBx isoform contains an Akt phosphorylation site at Ser31 and functions as an anti-apoptotic protein (designated as HBx-S31), whereas the other isoform lacks an Akt phosphorylation site and functions as an apoptotic protein (designated as HBx-L31). HBx-S31 can activate Akt, whereas HBx-L31 lacks this ability; the former enhances tumor growth, whereas the latter suppresses tumorigenesis. This finding shows that HBx can serve two functions (pro-apoptotic and anti-apoptotic) through different isoforms; HBx with Ser31 induces Akt signaling^31^. HBx-activated Akt phosphorylates the downstream target glycogen synthase kinase 3β (GSK-3β), thereby stabilizing β-catenin and enhancing promoter recruitment and expression of cyclin D1 and Bcl-XL^32^. In addition, HBx-activated Akt promotes the IKKα nuclear translocation via phosphorylating its threonine-23. IKKα ubiquitination, which is enhanced by HBx and Akt, contributes to IKKα accumulation in the nucleus, thereby indicating the involvement of ubiquitination in the Akt-increased IKKα nuclear transport in response to HBx^32^. The proliferation and colony formation of HCC cells are suppressed by miR-132-mediated inhibition of the Akt-signaling pathway in miR-132-transfected cells, as revealed by data from recent studies. Such data also revealed that HBx can repress the expression of miR-132 by DNA methylation^33^. The oncogenic cooperation between HBx and Akt may be important for cell proliferation, abrogation of apoptosis, and tumorigenic transformation of cells.

#### HBx and Wnt signaling pathway

Wnt pathway, an essential regulator of early embryonic development, contributes to somatic stem cell homeostasis. The canonical Wnt signal is initiated by the binding of Wnt ligands to the receptor Frizzled to activate Dishevelled (Dvl) proteins. Dvl transduces Wnt signal by suppressing the degradation of β-catenin via the adenomatosis polyposis coli (APC)/Axin/GSK3β destruction complex. The accumulation of β-catenin in the cytosol leads to its translocation into the nucleus, where the transcription of Wnt target genes is initiated^34^. The dysregulation of the Wnt pathway produces an improperly decayed β-catenin and an elevated β-catenin level, thereby hyper activating the transcription of cyclin D, VEGF, and c-MYC to subsequently promote the development and progression of HCC^35^. The increased expression of β-catenin in the cytoplasm or nucleus occurs in 50% to 70% of patients with HCC. Currently available studies showed that HBx competitively binds to APC to displace β-catenin from its degradation complex, resulting in β-catenin upregulation in the nucleus and the activation of Wnt signaling; thus, malignant transformation is induced^35^. In addition, the ectopic expression of HBx with Wnt-1 can activate Wnt/β-catenin signaling in Huh7 cells by stabilizing cytoplasmic β-catenin. Furthermore, the stabilization of β-catenin by HBx can be achieved by suppressing GSK-3 activity via the activation of Src kinase^36^. HBV-related HCC predominantly occurs in men; HBx is involved in this disparity between sexes by enhancing the transcriptional activity of the androgen receptor (AR) gene in a ligand-dependent manner. HBx can also enhance the AR transcriptional activity via c-Src and GSK-3β^37^. Thus, HBx is important in the activation of Wnt/β-catenin signaling in hepatocarcinogenesis.

#### HBx and signal transducer and activator of transcription (STAT)

STAT pathway serves a crucial function in the regulation of many key cellular processes, such as survival, proliferation, and differentiation. In the liver, the activation of STAT proteins, including STAT3 and STAT5, is critical for anti-viral defense against hepatitis viral infection and for controlling injury, repair, inflammation, and tumorigenesis^38^. In particular, HBx specifically upregulates the expression of tyrosine phosphorylation and increases the kinase activity of Jak1 through protein–protein interaction with Jak1. HBx induces the Jak–STAT signaling pathway^39^. The expression levels of p-STAT3 are positively correlated with those of the HBx expression. HBx can activate the Twist promoter by activating STAT3, which promotes the EMT occurrence in liver cells^40^. Previous studies revealed atypical activity. These observations emphasize the active role of HBx in hepatocarcinogenesis, particularly in the dysregulation of STAT signaling.

#### HBx and NF-κB

HBx is an activator of the transcription factor NF-κB, which is the first HBx-responsive motif to be identified^6^. HBx-mediated regulation of the heat shock protein gp96 expression requires an NF-κB cis-regulatory element in the promoter of gp96. HBx promotes the binding of NF-κB to the gp96 promoter^41^. Moreover, HBx directly regulates Notch1 signaling, which cross-talked with the NF-κB pathway [p65, p50, and IκB kinase α (IKKα)]. The downregulation of Notch1 decreases the binding of p65 to its target gene promoter, reduces NF-κB expression, and enhances IKKα expression^42^. Tumor necrosis factor (TNF)-α, which is an NF-κB signaling activator, induces the accumulation of HBx in cells by increasing protein stability, which is mainly caused by the reduction of proteasomal degradation. The effects of TNF-α on HBx protein stability are mediated via the activation of NF-κB effector kinases, such as IKKα, IKKβ, and p65, thereby indicating a positive feedback loop between HBx and NF-κB signaling pathways^43^. The ability of NF-κB, to mediate apoptosis is inhibited by HBx-expressing cells. The regulation of NF-κB mediated by HBx serves a central function in HBV-related HCC.

## Functions of HBx in hepatocarcinogenesis

### HBx enhances cell transformation

The hepatocarcinogenic effect of HBx on HBx-transgenic mice has been investigated. *HBx* gene expression can disrupt the normal cell growth in transgenic mice^44^. Studies suggested that HBx-transgenic mouse cells are characterized by malignant transformation^45^. Moreover, HBx protein exerts a strong growth arrest on hepatocytes and an imbalanced cell cycle progression, resulting in abnormal cell death; this condition is accompanied by severe fat accumulation and impaired glycogen storage in the HBx-transgenic livers^46^. HBx protein also blocks the G1/S transition of the hepatocyte cell cycle progression and causes liver functionality failure and cell death in the regenerating liver of HBx-transgenic mice^46^. The stable HBx transfection results in a malignant phenotype in the engineered cells *in vivo* and *in vitro*. HBx can increase the transcription of NF-κB, AP-1, and survivin to upregulate the expression levels of c-Myc and survivin. Abnormal centrosome duplication and activated human telomerase reverse transcriptase are responsible for the transformation^47^. The aberrant high expression of the *HBx* gene in normal cells strongly affects cell cycle progression and growth advantage, which promotes cell transformation^47^. HBx affects cellular fate by shifting the balance toward cell survival, probably causing loss of apoptotic functions or directly contributing to oncogenesis by gain of transforming functions. Therefore, HBx contributes to oncogenesis by stimulating cell proliferation. These studies indicate that HBx may function as an activator of hepatocarcinogenesis.

### HBx and apoptosis

Programmed cell death (apoptosis) is necessary for the elimination of redundant, damaged, and virally infected cells. Generally, proteins regulate apoptosis in three ways, as follows: as effectors and initiators of apoptosis, as inducers and suppressors of apoptosis, and as intermediate proteins^2^. HBx can regulate apoptosis through its action on caspases (cysteine–aspartate specific proteases), the mitochondria, and SIRT.

### HBx and cysteine aspartate specific proteases (caspases)

Caspases are a group of cysteine-containing proteases responsible for cellular maturation and reconstruction, number and quality of cell regulation, and apoptosis initiation of old cells or those that cannot perform their normal roles^48^. The apoptosis induced by the ectopic expression of HBx is associated with the alterations in mitochondrial membrane and caspases. The inhibition of Notch1 markedly promotes the apoptosis of LO2–HBx cells (stably transfected with HBx) via the caspase-9–caspase-3 pathway, whereas HBx can significantly improve the activity of Notch1^49^. Recent research provided *in vitro* and *in vivo* evidence that HBx can enhance the susceptibility of hepatocytes toward oxidative stress-induced apoptotic killing by accelerating the loss of Mcl-1 protein, which is mainly caspase-3 dependent^50^. This effect is an important factor in controlling HBx-related apoptosis.

#### HBx and the mitochondria

HBV infection alters mitochondrial metabolism. The selective removal of damaged mitochondria is important for maintaining mitochondrial and cellular homeostasis. Raf serine/threonine kinases 1 (Raf-1) can translocate to the mitochondria, thereby protecting the cells from stress-mediated apoptosis. Recent studies showed that HBx stimulates the mitochondrial translocation of Raf-1, thereby contributing to the anti-apoptotic effect^51^. In addition, HBV induces the perinuclear clustering of the mitochondria and triggers the mitochondrial translocation of dynamin-related protein by stimulating its phosphorylation at Ser616, leading to mitochondrial fission. HBx protein serves a central function in promoting aberrant mitochondrial dynamics when expressed alone or in the context of the viral genome^52^. Previous evidence indicated that HBx-expressing cells have an elevated mitochondrial calcium uptake. The increased mitochondrial calcium uptake mediated by HBx can sustain higher cytosolic calcium and stimulate HBV replication to accelerate the development of HCC^53^. We speculate that the altered mitochondrial dynamics associated with HBx contributes to mitochondrial injury and HCC pathogenesis.

#### HBx and programmed cell death 4 (PDCD4)

PDCD4 was originally identified as a tumor-related gene in humans. PDCD4 is expressed ubiquitously in normal tissues, and the highest level is found in the liver. PDCD4 is a proapoptotic molecule involved in the TGF-β1-induced apoptosis in human HCC cells and a possible tumor suppressor in hepatocarcinogenesis^54^. PDCD4 is downregulated in clinical HCC specimens, and such downregulation is correlated with HBx^9^. The mechanisms underlying PDCD4 downregulation by HBx are partly through miR-21^55^. These data elucidate the anti-apoptotic function of the HBx viral protein and its contribution to HCC.

### HBx and immune system

To successfully detect and eliminate invading pathogens by discriminating self from non-self, the mammalian immune system develops mechanisms, which comprise two distinct components, namely, the innate immunity and the adaptive immunity. In most multicellular organisms, the highly conserved innate immune system provides the first line of defense to limit infection by detecting pathogens using germline-encoded proteins^56^. The adaptive immunity, which is present only in vertebrates, detects non-self by recognizing peptide antigens through the receptors expressed on the surface of B and T cells^57^. HBx has different strategies for affecting immune system to promote the initiation and progression of HCC.

#### HBx and innate immune

The innate immune system is characterized by the production of type I interferon-α/β (IFN-α/β) cytokines and the activation of NK cells; this immune system has important roles in detecting and eliminating invading pathogens. Previous studies reported that HBx interacts with mitochondrial antiviral signaling (MAVS) and promotes the degradation of MAVS through Lys136 ubiquitin in MAVS protein to prevent the induction of IFN-β^58^. Further analysis on clinical samples revealed that MAVS protein is downregulated in the HCCs of HBV origin; this downregulation is correlated with the increased sensitivities of the primary murine hepatocytes isolated from HBx knock-in transgenic mice upon vesicular stomatitis virus (VSV) infections^58^. In addition, experimental evidence revealed that IFN induction is blocked by HBV replication in HepG2.2.15 cells. This effect is partially due to HBx, which impairs the IFN-β promoter activation using Sendai virus and the components implicated in signaling by viral sensors^59^. Moreover, HBx–siRNAs significantly promote PKR activation, leading to the higher production of type I IFN^60^. All these studies suggest that HBx can attenuate the antiviral response of the innate immune system and then promotes hepatocarcinogenesis. These findings delineated some functions of HBx-induced immune dysfunction in the development of HBV-associated HCC.

#### HBx and adaptive immunity

CD8+ T cells contribute to the clearance of HBV infection, and the lack of CD8+ T cells may be among the major factors that cause chronic HBV infection and HCC development. HBx expression induces the low production of interferon-γ and the apoptosis of CD8+ T cells without affecting CD8+ T cell proliferation^61^. As critical component in the tumor microenvironment, immunocytes can affect tumor prognosis by affecting adaptive immunity^62^. Interleukin-12 (IL-12) is a disulfide-linked heterodimeric cytokine with potent immunostimulatory activity. Recent data revealed that co-expression of HBx and IL-12 can induce a massive accumulation of CD8+ T cells, which inhibits stromal cell growth, such as in vascular endothelial cells^62^. Other studies showed the apparent infiltration of CD8+ T lymphocytes in the tumor of an HBx-treated group. Moreover, flow cytometric analysis showed that the component of CD8+ T lymphocytes after HBx vaccination is larger than that of the control groups. Moreover, the depletion of CD8+ T lymphocytes showed a complete abrogation of the antitumor immune activity *in vivo*^63^. These findings strongly confirm that HBx-related adaptive immunity is very important in the development of HCC.

Overall, we summarized the mechanism of HBx in hepatocarcinogenesis. HBV-associated hepatocarcinogenesis can be viewed as a multi-factorial process, which includes direct and indirect mechanisms that may act cooperatively (**Table 1**). However, considering the numerous evidence implicating many different molecules and pathways in the development of HCC, the exact mechanisms by which HBx induces hepatocarcinogenesis remain ambiguous. Further research is required. The above mentioned data provide insights into the cell-transforming potential of HBx. HBx can interact with many transcription factors, regulatory RNAs, and various signaling transduction pathways. HBx is also involved in the modification of epigenetics. Additionally, HBx induces HBV-related HCC and can modulate apoptosis and the immune system. HBx serves a major function in cell survival and hepatocellular transformation during HBV infection. Biologically elucidating the significant activity of HBx may provide further insight into the role of HBx and may ultimately lead to the development of novel therapeutic strategies for managing HBV-related HCC and for the clinical therapy of HCC. The definition of HBx function *in vivo* should be the focus of future studies.

**Table 1:** Summary of HBx in hepatocarcinogenesis

Mechanisms of HBx-induced hepatocarcinogenesis
Regulatory noncoding RNAs
miRNAs
miR-148a activates mTOR^11^
miR-15b increases the levels of Globo H^12^
miR-205 increases the expression of ACSL1 and triglyceride^13^^,^^14^
miR-224 silences Smad4^15^
lncRNAs
HULC suppresses the expression of p18^16^
Dreh represses the expression of intermediate filament protein vimentin^17^
Epigenetic modiﬁcation
Methylation
Upregulates the expression of DNMT1 and DNMT3A^21^^-^^23^
Acetylation
Induces the expression of HDAC1^24^^-^^26^
Upregulates SIRT1^28^^,^^29^
Functions of HBx in HBx-related hepatocarcinogenesis
Signaling pathway
Akt
Stabilizes β-catenin^32^
Promotes IKKα nuclear translocation^33^
Wnt
Hyperactivates the transcription of cyclin D, VEGF, c-MYC, and AR^36^^,^^37^^,^^64^
Stat
Activates the Twist promoter^39^^,^^40^
NF-κB
Upregulates the heat shock protein gp96^41^
Enhances the expression of IKKα^42^^,^^43^
Cell transformation
Exerting strong growth arrest and imbalanced cell-cycle progression^45^^-^^47^
Apoptosis
Caspases
Inhibit Notch signaling^49^
Accelerate the loss of Mcl-1 protein^50^
Mitochondria
Stimulate the mitochondrial translocation of Raf-1^51^
Induce the perinuclear clustering of the mitochondria^52^
Increase the mitochondrial calcium uptake^53^
PDCD4
Induce TGF-β1-mediated apoptosis^9^^,^^55^
Immune system
Innate immune
Prevent the induction of IFN-β^58^^,^^59^
Adaptive immunity
Accumulate CD8+ T cells^62^
